# Impact of First- and Second-Generation Tyrosine Kinase Inhibitors on the Development of Graft-Versus-Host Disease in Individuals with Chronic Myeloid Leukemia: A Retrospective Analysis on Behalf of the Polish Adult Leukemia Group

**DOI:** 10.3390/biomedicines13010163

**Published:** 2025-01-11

**Authors:** Ugo Giordano, Agnieszka Piekarska, Witold Prejzner, Lidia Gil, Jan Maciej Zaucha, Joanna Kujawska, Zuzanna Dybko, Krzysztof Dudek, Sebastian Giebel, Jarosław Dybko

**Affiliations:** 1 Department and Clinic of Endocrinology and Internal Medicine, Wrocław University Hospital, 50-367 Wroclaw, Poland; 2 Department of Hematology and Transplantology, Medical University of Gdansk, 80-210 Gdansk, Poland; agnieszka.piekarska@gumed.edu.pl (A.P.); witold.prejzner@gumed.edu.pl (W.P.); jan.zaucha@gumed.edu.pl (J.M.Z.); 3 Department of Hematology and Bone Marrow Transplantation, Poznan University of Medical Sciences, 60-569 Poznan, Poland; lidia.gil@skpp.edu.pl (L.G.); joanna.kujawska@usk.poznan.pl (J.K.); 4 Faculty of Medicine, Wroclaw Medical University, 50-367 Wrocław, Poland; zuzanna.dybko@student.umed.wroc.pl; 5 Faculty of Mechanical Engineering, Wroclaw University of Science and Technology, Wybrzeze Wyspianskiego 27, 50-370 Wroclaw, Poland; krzysztof.dudek@pwr.edu.pl; 6 Department of Bone Marrow Transplantation and Oncohematology, Gliwice Branch, Maria Sklodowska-Curie National Research Institute of Oncology, 44-102 Gliwice, Poland; sebastian.giebel@io.gliwice.pl; 7 Department of Hematology and Cellular Transplantation, Lower Silesian Oncology Center, 53-413 Wroclaw, Poland; jaroslaw.dybko@dcopih.pl; 8 Department of Oncology and Hematology, Faculty of Medicine, Wroclaw University of Science and Technology, 50-370 Wroclaw, Poland

**Keywords:** allogeneic hematopoietic stem cell transplantation, chronic myeloid leukemia, tyrosine kinase inhibitors, imatinib, dasatinib, nilotinib

## Abstract

**Background**: The implementation of tyrosine kinase inhibitors (TKIs) in the treatment of chronic myeloid leukemia (CML) has brought a significant improvement in the prognosis for CML patients and a decrease in the number of patients requiring allogeneic hematopoietic stem cell transplantation (allo-HCT). Nevertheless, the impact of TKIs on allo-HCT outcomes has not been thoroughly explored. **Objectives**: The main endpoint of our research was to assess the impact of prior TKI treatment on acute graft-versus-host disease (aGvHD) and chronic graft-versus-host disease (cGvHD). **Methods**: In our retrospective analysis, we included 240 patients treated between 1993 and 2013 and divided them into three groups according to the therapy administered prior to haploidentical, matched-related, or matched-unrelated donor allo-HCT (imatinib group *n* = 41, dasatinib/nilotinib group *n* = 28, TKI-naïve group *n* = 171). **Results**: Both the cumulative incidence of aGvHD (*p* = 0.044) and cGvHD (*p* < 0.001) in individuals receiving second-generation TKIs (2G-TKIs) prior to allo-HCT were decreased compared to patients receiving no TKIs or imatinib (IMA) (40.7% vs. 61.4% vs. 70.7%, *p* = 0.044; 25.0% vs. 76.4% vs. 51.2%, *p* < 0.001, respectively). In the case of the 2G-TKI cohort, the number of low-grade aGvHD and cGvHD was significantly lower compared to the IMA and TKI-naïve groups (*p* = 0.018, *p* = 0.004; *p* < 0.001 versus TKI-naïve, respectively). In terms of 3-year overall survival (OS), there were no important variations between TKI-naïve, IMA, and 2G-TKI (55% vs. 49.9% vs. 69.6%, *p* = 0.740). **Conclusions**: The results of our study suggest that TKI treatment prior to allo-HCT may have a protective impact on immune-mediated outcomes.

## 1. Introduction

Tyrosine kinase inhibitors (TKIs) have become a widely accepted therapeutic approach for patients diagnosed with chronic myeloid leukemia (CML) ever since the introduction of the first TKI in the late 1990s, that is, imatinib mesylate [1]. The groundbreaking outcomes of the IRIS trial further solidified their pivotal role in revolutionizing the management of this hematopoietic malignancy [2]. Second-generation TKIs (2G-TKIs), especially dasatinib and nilotinib, have demonstrated highly promising results in cases of imatinib resistance or intolerance, yielding a long-term overall survival (OS) rate exceeding 70% [3,4]. Moreover, in newly diagnosed CML patients, the 5-year cumulative probability of achieving a major molecular response (MMR) exceeds 75%, markedly surpassing the corresponding rate attained with imatinib [5,6]. Although TKIs have proven to be effective, allo-HCT continues to be a viable and potentially curative alternative for patients who do not achieve long-lasting responses to TKI-based therapies or for those with advanced-stage disease [7,8].

The significance of allo-HCT in achieving long-term survival and potential cure for patients diagnosed with chronic phase CML (CML-CP) was well established before the advent of TKIs [9,10]. The concept of a graft-versus-leukemia effect in CML gained support from studies demonstrating that donor lymphocyte infusion could effectively salvage relapsed disease after allo-HCT [11,12]. However, the number of performed allo-HCTs for CML in the first chronic phase (CP1) has substantially diminished following the introduction of TKIs [13]. Currently, the majority of patients referred to allo-HCT are those considered at high risk, that is, at disease stages beyond CP1, or those that have experienced treatment failure with TKIs [14,15]. According to the latest update on CML management [16], allo-HCT has been suggested to be a viable option in CML-CP with resistance to 2GTKI therapy with no guiding mutations or in cases of a potential T315I mutation after either ponatinib or asciminib treatment on a trial basis [17,18]. Despite a significant reduction in the incidence of CML-BP with the introduction of TKIs compared to the pre-TKI era [19], responses to TKI treatment in CML-BP patients are of a temporary character, and their prognosis remains poor despite advancements in drug development [20]. Hence, allo-HCT remains the sole curative option for this subgroup of patients, and TKIs may offer a therapeutic window that allows for the possibility of allografting [20]. Currently, individuals with CML accelerated phase (CML-AP) or CML blast phase (CML-BP) might undergo TKI treatment in order to lower the CML burden (preferably with newer generations of TKIs such as ponatinib or dasatinib) and should afterwards be considered for early allo-HCT [21].

According to previous research, the use of imatinib does not negatively impact transplant outcomes, including acute and chronic graft-versus-host disease (aGvHD/cGvHD) [22,23,24]. Similarly, trials designed to evaluate 2G-TKI treatment prior to allo-HCT have not provided any evidence of 2G-TKIs having a harmful effect on the post-transplantation period [25,26]. Also, prior exposure to TKIs could potentially result in patients having better allo-HCT outcomes if transplantation is performed in a better molecular response status [24,27]. Considering the numerous roles of kinases in the GvHD pathophysiology, it was supposed that the administration of TKIs could represent a potentially effective approach for regulating the activation of B and T cells, resulting in the effective treatment of GvHD [28]. It should be noted that increased direct drug toxicity [29] or immune dysfunction [30] may also limit the success of allo-HCT, considering that each TKI has multiple off-target effects.

This retrospective study on behalf of the Polish Adult Leukemia Group (PALG) aims to compare GvHD and survival outcomes of allo-HCT of three groups comprising a cohort of patients from the pre-TKI era and individuals who underwent either first-generation TKI (1G-TKI) or 2G-TKI treatment prior to transplantation, all of whom suffered from CML.

## 2. Materials and Methods

### 2.1. Patients

This analysis is focused on CML patients treated with allogeneic stem cell transplantation from haploidentical, matched-related, or matched-unrelated donors (MRD/MUD) in different treatment eras. We analyzed three groups of patients: TKI-naïve, so-called historic group, collecting patients transplanted before the imatinib era; the second one (IMA) with patients transplanted after imatinib failure before second-generation TKIs became widely available; and the last one (2G-TKI), transplanted after second-generation TKIs failure (dasatinib or nilotinib), used as a second-line treatment after imatinib failure. The patients were treated in several PALG-associated centers between 1993 and 2013. The inclusion criteria comprised patients suffering from CML who eventually underwent allo-HCT without the administration of TKIs or with prior 1G-TKI or 2G-TKI treatment. Patients whose therapy did not include allo-HCT or who had other hematological malignancies were excluded from this study. In the statistical analysis, there were taken variables such as sex, patients’ age, disease duration before transplant, using IFNα and TKIs during the treatment and its duration, CML phase at the day of transplant, conditioning regimen, application of GvHD prophylaxis, presence and grade of acute and chronic GvHD after the procedure of allo-HCT, evaluation in the Karnofsky scale, AB0 mismatch, transplant risk category (using the Grathwohl scale), and donor CMV status. All the patients underwent haploidentical, MRD, or MUD allo-HCT with reduced intensity or myeloablative conditioning regimens (RIC/MAC). GvHD prophylaxis followed standard protocols, which comprised cyclosporine A (CsA), methotrexate (MTX), and, in cases of a MUD, also anti-thymocyte globulin (ATG). Patient-, donor-, and transplant-related characteristics can be found in Table 1 and Table 2.

### 2.2. Definitions

CML chronic and advanced phases were diagnosed according to ELN criteria [31,32]. Acute graft versus host disease diagnosis and grading was based on the 1994 consensus [33] with an update of the MAGIC consortium [34] regarding overall clinical grade calculation (based upon the most severe target organ involvement): grade 0—no stage 1–4 of any organ; grade 1—stage 1–2 skin without liver, upper GI, or lower GI involvement; grade 2—stage 3 rash and/or stage 1 liver and/or stage 1 upper GI and/or stage 1 lower GI; stage 3—stage 2–3 liver and/or stage 2/3 lower GI, with stage 0–3 skin and/or stage 0–1 upper GI; stage 4—stage 4 skin, liver, or lower GI involvement, with stage 0–1 upper GI. The diagnosis and global scoring of chronic graft versus host disease came from the National Institute of Health 2005 criteria [35], but we had to combine cases scored 2 and 3 in one group to standardize the grading in all three groups. The historic group grading was based on traditional Seattle 1980 criteria [36] referring to “limited” and “extensive” chronic GvHD cases. To make the analysis clear, we put NIH 2005 scores 2 and 3 cases (from the “new era” transplantations) and Seattle “extensive” cases in one advanced group—2. NIH score 1 and “limited” Seattle cases were put in one group as well—1.

### 2.3. Study Endpoints

The main objective of this retrospective analysis is to assess the impact of first-generation TKI and second-generation TKI treatment on the development of aGvHD and cGvHD in individuals with CML undergoing allo-HCT. The secondary outcomes included 3-year overall survival (OS).

### 2.4. Statistical Analysis

Statistica v.13.3 (TIBCO Software Inc., Palo Alto, CA, USA) was used for statistical analysis. Verification of normality of quantitative variables was performed using the Shapiro–Wilk test. Due to the absence of normal distribution characteristics or heterogeneity of variance, the statistical significance of differences between the three groups was assessed using the non-parametric Kruskal–Wallis test. The significance of differences in frequencies for qualitative variables was calculated using the chi-square test. The Kaplan–Meier estimator was used to estimate survival probabilities. The significance of differences between survival curves in the three groups was verified by the chi-square test. Statistical test results were deemed statistically significant when the *p*-value was <0.05. In multivariate analysis, considering that aGvHD and cGvHD are binary variables, logistic regression was employed. The results of multivariate analysis are presented in Table 3.

## 3. Results

### 3.1. Patient-, Disease-, and Transplantation-Related Characteristics

The characteristics of all individuals divided into three subgroups—TKI-naïve (*n* = 171), IMA (*n* = 41), and 2G-TKI (*n* = 28)—are presented in Table 1 and Table 2. The 2G-TKIs used prior to allo-HCT were either dasatinib or nilotinib, while the 1G-TKI was imatinib. The disproportion in the number of individuals in each cohort is a consequence of the introduction of TKIs, which drastically reduced the number of performed allo-HCTs. The characteristics in the three groups were comparable with regard to patient sex and donor sex/age. The individuals in the 2G-TKI group were significantly older (37 vs. 35 vs. 48 years of age, *p* < 0.001), likely due to the refinement of CML therapy resulting in a longer time-to-transplant period. The ratios of MRD/MUD allo-HCTs were 75.5%/21.6% for TKI-naïve, 34.1%/65.9% for IMA, and 35.7%/64.3% for 2G-TKI (*p* < 0.001), with either BM or PBSC being the source of stem cells (58.5%/41.5%, 46.3%/53.7%, 14.3%/85.7%, *p* < 0.001, respectively). On the day of the transplant, most individuals in every cohort were in CP1: 88% vs. 37.9% vs. 71.4%, *p* = 0.002 for TKI-naïve, IMA, and 2G-TKI, respectively. Of note, there were significantly more patients in their second or next CP at the moment of allo-HCT in the IMA group (3.6% vs. 32.4% vs. 0%), which might have influenced survival outcomes. Patients who received allo-HCT in CML-BP or CML-AP were highly treatment-resistant mutated cases who proceeded to salvage allo-HCT. Out of the 28 patients in the 2G-TKI group, 10 individuals manifested imatinib intolerance, 12 individuals had bcr-abl-independent TKI resistance, and 6 individuals had bcr-abl mutations. The number of individuals receiving RIC conditioning was significantly higher in 2G-TKI (11.8% vs. 17.1 % vs. 46.4%, *p* < 0.001). Also, the median CD34+ count was comparable among the three cohorts (4.0 × 10^6^/kg vs. 5.5 × 10^6^/kg vs. 4.0 × 10^6^/kg, *p* = 0.543, respectively). The median follow-up time was 41 months for TKI-naïve, 19 months for IMA, and 30 months for 2G-TKI (*p* < 0.001).

### 3.2. Acute and Chronic Graft-Versus-Host Disease

**Figure 1:**
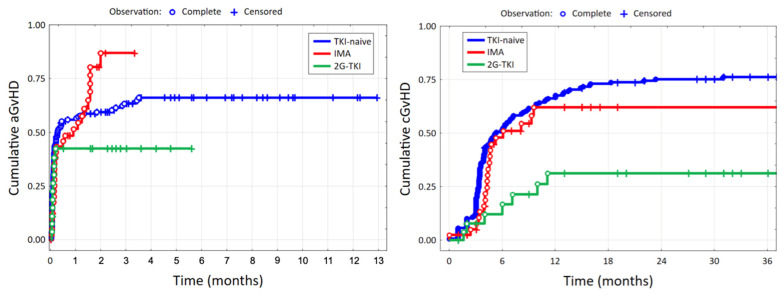
Impact of 1st generation and 2nd generation TKIs on the cumulative HR of aGvHD (12 months) and cGvHD (36 months).

**Figure 2:**
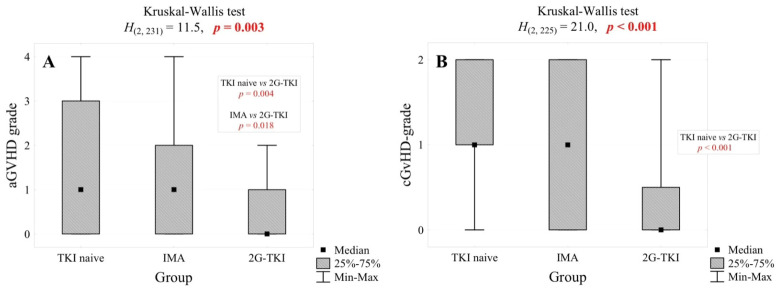
Impact of 1st generation and 2nd generation TKIs on the grade of aGvHD (**A**) and cGvHD (**B**).

Data about aGvHD and cGvHD can be found in Table 2 and Figure 1 and Figure 2A,B. Out of the 240 patients included in the analysis, GvHD grade assessment was performed in 231 individuals for aGvHD and 226 for cGvHD. The outcomes of both the cumulative incidence of aGvHD and cGvHD were the least favorable in the TKI-naïve and IMA cohorts compared to 2G-TKI (61.4% vs. 70.7% vs. 40.7%, *p* = 0.044; 76.4% vs. 51.2% vs. 25%, *p* = 25.0%, *p* < 0.001, respectively). Also, the 2G-TKI cohort yielded significantly more low-grade aGvHD compared to TKI-naïve and IMA (*p* = 0.004, *p* = 0.018, respectively) and cGvHD in contrast to TKI-naïve (*p* < 0.001). By multivariate analysis, a female donor setting (HR 2.17, 95% CI 1.15–4.08, *p* = 0.017) and ATG-based GvHD prophylaxis (HR 2.80, 95% CI 1.15–6.79, *p* = 0.023) had an adverse impact on aGvHD incidence. Also, CML accelerated phase (HR 3.13, 95% CI 1.05–9.37, *p* = 0.041) and female donor (HR 2.43, 95% CI 1.26–4.69, *p* = 0.009) were predictors of cGvHD. RIC conditioning lowered the occurrence of cGvHD (HR 0.24, 95% CI 0.11–0.51, *p* < 0.001). The median day of aGvHD and cGvHD onset was comparable among the three groups (36 vs. 44 vs. 32, *p* = 0.894; 139 vs. 131 vs. 257, *p* = 0.625, respectively).

### 3.3. Survival Outcomes

**Figure 3:**
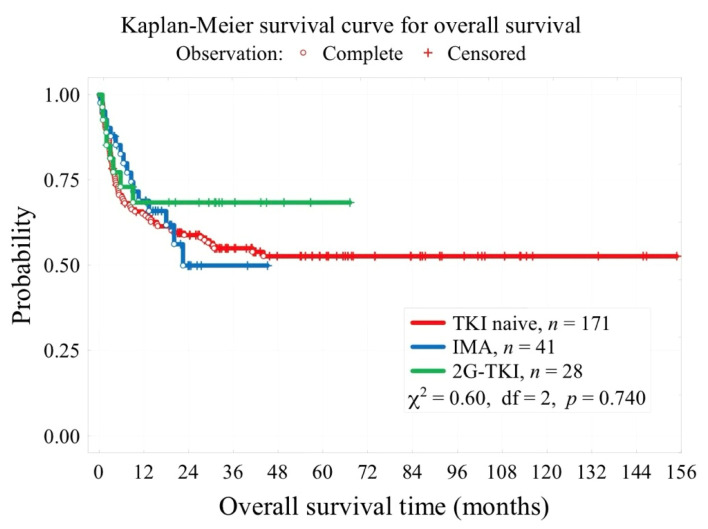
Impact of 1st generation and 2nd generation TKIs on OS probability.

Survival outcomes are shown in Table 4 and Figure 3. The 3-year OS was comparable between the three cohorts (55% vs. 49.9% vs. 69.6%, *p* = 0.740).

## 4. Discussion

The introduction of TKIs had a pivotal role in the changing landscape of CML-CP management [37]. In the frontline treatment of CML-CP, imatinib and the three 2G-TKI formulations (bosutinib, dasatinib, and nilotinib) yield comparable survival outcomes. However, second-generation TKIs can induce deep molecular response (DMR) more rapidly, potentially shortening the time to achieve treatment-free remission (TFR) [38]. Taking into account TKI-related toxicities, there is an ongoing debate about the most appropriate moment for considering the discontinuation of TKI therapy. Most experts currently lean towards sustaining TKI treatment in the absence of adverse effects, as it was proved that ceasing TKIs after 2 years of DMR yields 3-year TFR rates of 40–50% [39,40], while discontinuation after achieving a DMR for at least 5 years results in a 5-year TFR of over 80% [41]. In spite of these promising results, the current recommendations indicate that allo-HCT should be considered in case of treatment failure or intolerance to at least one 2GTKI or in case of a T315I mutation—after a trial therapy with ponatinib or ascimib [17,18]. Also, allo-HCT should be considered in CML-AP and CML-BP after lowering the CML burden through a new-generation TKI [24,27]. In our retrospective study, we sought to assess the influence of IMA and 2G-TKI therapy prior to allo-HCT compared to a historic group from the pre-TKI era on transplant-related toxicity, that is, the occurrence of aGvHD and cGvHD, as well as analyze survival outcomes.

Our subgroup analysis revealed that individuals from the 2G-TKI cohort experienced aGvHD significantly less frequently, and if it occurred, its grade was lower compared to TKI-naïve and IMA (40.7% vs. 61.4% vs. 70.7%, *p* = 0.044; *p* = 0.018 vs. TKI-naïve, *p* = 0.004 vs. IMA, respectively, as shown in Figure 1 and Figure 2A). We demonstrated similar results for cGvHD, with 2GTKIs guaranteeing more favorable outcomes both with regard to overall cGvHD incidence (25% vs. 76.4% vs. 51.2%, *p* < 0.001) and cGvHD grade (2GTKI vs. TKI-naïve *p* < 0.001, Figure 2B). No differences were found with regard to the day of onset of aGvHD and cGvHD (day +32 vs. day +36 vs. day +44, *p* = 0.849; 139 vs. 131 vs. 257, *p* = 0.625, respectively). According to the results of multivariate analysis, neither myeloablative conditioning (MAC) nor MUD nor the recipient’s age influenced the occurrence of GvHD. Among the retrievable literature, four studies included a comparison of the influence of prior IMA therapy on post-transplant outcomes with historical groups [22,23,42,43], and one performed a similar analysis but with first-, second-, and third-generation TKIs [44]. In terms of aGvHD incidence, none of the aforementioned papers demonstrated statistically significant differences between the TKI-treated and TKI-naïve cohorts, regardless of the TKI generation [22,23,42,43,44]. Discrepancies have been found concerning cGvHD despite most studies corroborating that TKI treatment does not have an impact on its incidence [42,43,44]. In the research by Oehler et al. [23] analyzing allo-HCT outcomes of 145 individuals administered IMA and 231 from the historical groups, IMA therapy resulted in a significantly lower hazard of cGvHD (HR = 0.33, 95% CI 0.22–0.48, *p* ≤ 0.001). A similar observation was made in a trial by Deininger et al. [22], which consisted of a comparison of 70 patients with CML and 21 with Ph+ ALL receiving pre-transplant IMA with historical controls identified in the EBMT database. Individuals exposed to IMA prior to allo-HCT yielded a significantly lower incidence of cGvHD in comparison to control (36.7% vs. 58.8%, *p* = 0.03), which was confirmed by multivariate analysis (OR = 0.44, *p* = 0.027) [22].

Given the multifactorial nature of GvHD pathogenesis, we made an effort to account for various contributing factors in our analysis, complementing a comprehensive review of the available literature. PBSC as a source of stem cells has been previously associated with a higher incidence of aGvHD and cGvHD [45] and cGvHD alone [46,47]. In our analysis, the 2G-TKI group had the highest percentage of PBSC vs. BM in comparison with TKI-naïve and IMA (85.7% vs. 41.5% vs. 53.7%, *p* < 0.001; 2G-TKI vs. IMA *p* = 0.02, 2G-TKI vs. TKI-naïve *p* = 0.001). Furthermore, RIC conditioning was significantly more frequent in the 2G-TKI group (11.8% vs. 17.1% vs. 46.4%, *p* < 0.001). There are comparative studies confirming reduced overall incidence of aGvHD grades 2–4 in patients conditioned with RIC [48,49], but none of them has been able to show a difference in terms of cGvHD incidence depending on the type of conditioning regimen. Moreover, recent studies surprisingly revealed RIC to be a factor increasing the incidence of cGvHD [50], which, however, is in contradiction to the results of our multivariate analysis, as it revealed that RIC conditioning lowered the occurrence of cGvHD (HR 0.24, 95% CI 0.11–0.51, *p* < 0.001). Taking into account these results, we could assume that RIC conditioning might have had a positive impact on aGvHD occurrence in the 2G-TKI cohort, but it is not certain whether it should be enrolled in the list of cGvHD protective agents. The recent development of more effective GVHD prophylactic regimens should also be considered, particularly when analyzing data from patients treated in more recent years (IMA and 2G-TKI groups). Moreover, the use of IFNα, particularly when administered in a short period prior to allo-HCT, might be one of the reasons for the higher occurrence of aGvHD in the IMA group (61.4% for TKI-naïve vs. 70.7% for IMA vs. 40.7% for 2G-TKI, *p* = 0.044), as a significant number of patients in this group received IFNα compared to other cohorts (14.8% for TKI-naïve vs. 47.1% for IMA vs. 0% for 2G-TKI, *p* < 0.001). Cox logistic regression demonstrated that a female donor setting (HR 2.17, 95% CI 1.15-4.08, *p* = 0.017) and ATG-based GvHD prophylaxis (HR 2.80, 95% CI 1.15-6.79, *p* = 0.023) negatively influenced aGvHD incidence. As for cGvHD, CML-AP (HR 3.13, 95% CI 1.05-9.37, *p* = 0.041) and female donor (HR 2.43, 95% CI 1.26-4.69, *p* = 0.009) were predictors of its occurrence. The number of female donors was comparable among the three groups, with, however, significant differences in the ratio of CML-AP (8.4% for TKI-naïve vs. 21.6% for IMA vs. 14.3% for 2G-TKI, *p* < 0.001). Of note, a significant number of patients received IFNα prior to allo-HCT compared to other cohorts (14.8% vs. 47.1% vs. 0%, *p* < 0.001), which might be the cause of the high occurrence of aGvHD in that group (61.4% vs. 70.7% vs. 40.7%, *p* = 0.044).

We did not demonstrate important variations of 3-year OS between TKI-naïve, IMA, and 2G-TKI (55% vs. 49.9% vs. 69.6%, *p* = 0.740), but we found it surprising that the IMA group manifested a tendency towards a lower 3-year OS compared to the TKI-naïve one. This observation is most likely the result of a substantial variance in the number of individuals in the second or next CP at the moment of allo-HCT (3.6% vs. 32.4% vs. 0% for TKI-naïve, IMA, and 2G-TKI, respectively), which might have negatively impacted survival outcomes in the IMA subgroup. Poor 3-year OS in IMA and 2G-TKI could be related to a substantial number of patients in CML-BP for 2G-TKI (0% vs. 8.1% vs. 14.3% for TKI-naïve, IMA, and 2G-TKI, respectively) and CML-AP in IMA (8.4% vs. 21.6% vs. 14.3% for TKI-naïve, IMA, and 2G-TKI, respectively). In a retrospective study by Shen et al. [43], the use of IMA was also associated with a significantly higher 0.5-year transplant-related mortality (27.8% vs. 5.9%, *p* = 0.001) compared to no prior TKI therapy. It should be noted that, like in our study, it is probable that this finding could be related to a relevant difference in disease stage at the moment of allo-HCT between the two groups (total BC + AP + CP2 + CP3 in 47.2% for IMA vs. 8.6% TKI-naïve, *p* = 0.001) [43]. The other papers found that long-term survival outcomes were not affected either by the use of IMA prior to allo-HCT [22,23,42,43] or first-, second-, and third-generation TKIs [44].

Our study has some limitations, such as its retrospective design and a relatively short follow-up time in the IMA and 2G-TKI groups. Also, not all the patients who developed GvHD could have been assessed utilizing the same tools, due to the need for standardization (as described in the Materials and Methods section). Finally, the discrepancies in cohort sizes, especially the smaller 2G-TKI group, lead to significant heterogeneity of certain parameters among the three cohorts, thus limiting the quality of evidence. We believe that this is a result of the sequential introduction and widespread use of imatinib, which drastically decreased the need for allo-HCT. The development of novel GvHD prophylactic regimens should also be considered.

## 5. Conclusions

Despite the aforementioned caveats to our study, the results we obtained confirm that TKI treatment prior to allo-HCT does not negatively impact the post-transplantation outcomes, including aGvHD and cGvHD. Our analyses suggest that 2G-TKI therapy could significantly lower the cumulative incidence of cGvHD and aGvHD as well as their grade in case of their occurrence. Also, we demonstrated no variations in terms of 3-year OS between the three cohorts. In our view, this analysis remains relevant despite the prevalent use of TKIs, which have drastically changed the landscape of CML therapy in the past decades. Unlike CML-CP patients, who often proceed to allo-HCT following resistance or intolerance to second-generation TKI therapy or after a trial therapy with ascimib or ponatinib in the case of T315I mutation, allo-HCT in CML-AP or CML-BP should be considered after lowering the CML burden with new-generation TKIs [17,18]. This study offers an analysis of the outcomes of allo-HCT for these subgroups of patients.

## Figures and Tables

**Table 1:** Patient-, donor-, and transplant-related characteristics. Abbreviations: IMA—imatinib, 2G-TKI—2nd generation tyrosine kinase inhibitor, BM—bone marrow, PBSC—peripheral blood stem cells, CML—chronic myeloid leukemia, HU—hydroxyurea, IFNα—interferon-alfa, RIC—reduced-intensity conditioning, TBI—total body irradiation, ATG—anti-thymocyte globulin, transplant risk category (Karnofsky scale)—tool used to evaluate the functional status of patients, ranging from 0 (death) to 100 (normal function), AB0 mismatch—incompatibilities in blood group antigens between donor and recipient, transplant risk category (Grathwohl scale)—grading system used to predict transplantation outcomes based on patient-specific factors. Statistically significant results are highlighted in bold. Note: PTCy in haploidentical transplantation was introduced in Poland in the years 2005–2007.

	TKI-Naïve *n* = 171		IMA *n* = 41		2G-TKI *n* = 28		*p*-Value
Patient sex, *n* (%)							0.211
Male	100	58.8%	26	63.4%	12	42.9%	
Female	71	41.2%	15	36.6%	16	57.1%	
Patient age (years), Me [Q1; Q3]	37 [28; 44]		35 [26; 40]		48 [33; 57]		**<0.001**
Donor sex, *n* (%)							0.766
Male	101	58.7%	26	63.4%	0	0.0%	
Female	70	41.3%	15	36.6%	28	100%	
Donor age (years), M ± SD	37.0 ± 10.4		37.6 ± 11.3		-		0.769
Type of donor, *n* (%)							**<0.001**
Matched-related donor	129	75.5%	14	34.1%	10	35.7%	
Matched-unrelated donor	37	21.6%	27	65.9%	18	64.3%	
Haploidentical donor	5	2.9%	0	0.0%	0	0.0%	
Source of stem cells, *n* (%)							**<0.001**
BM	100	58.5%	19	46.3%	4	14.3%	
PBSC	71	41.5%	22	53.7%	24	85.7%	
Transplant risk category, Me [Q1; Q3]	2 [1; 3]		3 [3; 4]		-		1.000
Median CD34+ count ×10^6^/kg, Me [Q1; Q3]	4.0 [2.7; 5.7]		5.5 [2.2; 7.1]		4.0 [3.6; 4.7]		0.543
Donor-positive CMV status, *n* (%)	99	77.3%	21	51.2%	NA		**0.006**
CML phase at day of transplant, *n* (%)							**<0.001**
Chronic phase	147	88.0%	14	37.9%	20	71.4%	
Accelerated phase	14	8.4%	8	21.6%	4	14.3%	
Blast crisis phase	0	0.0%	3	8.1%	4	14.3%	
Second/next chronic phase	6	3.6%	12	32.4%	0	0.0%	
HU 1 year before transplant	140	98.6%	14	66.7%	NA		**<0.001**
IFNα (yes), *n* (%)	22	14.8%	16	47.1%	0	0.0%	**<0.001**
RIC (yes), *n* (%)	20	11.8%	7	17.1%	13	46.4%	**<0.001**
High dose TBI (yes), *n* (%)	6	3.5%	11	28.9%	1	3.6%	**<0.001**
ATG in conditioning regimen	48	28.1%	27	71.1%	NA		**<0.001**

**Table 2:** Patient-, donor-, and transplant-related characteristics (continuation). The comment “for (number) pt.” means the number of patients for whom data were available. Abbreviations: IMA—imatinib, 2G-TKI—2nd generation tyrosine kinase inhibitor, aGvHD—acute graft-versus-host disease, cGvHD—chronic graft-versus-host disease, transplant risk category (Karnofsky scale)—tool used to evaluate the functional status of patients, ranging from 0 (death) to 100 (normal function). Statistically significant results are highlighted in bold.

	TKI-Naïve *n* = 171		IMA *n* = 41		2G-TKI *n* = 28		*p*-Value
aGvHD day, Me [Q1; Q3]	36 [24; 54]		44 [23; 59]		32 [24; 49]		0.894
cGvHD day, Me [Q1; Q3]	139 [103; 250]		131 [120; 151]		257 [132; 317]		0.625
aGvHD (yes), *n* (%)	for 163 pt.				for 27 pt.		**0.044**
	100	61.4%	29	70.7%	11	40.7%	
aGvHD grade, *n* (%)	for 163 pt.				for 27 pt.		**0.002**
0	63	38.7%	12	29.3%	16	59.3%	
1	25	15.3%	9	22.0%	9	33.3%	
2	33	20.2%	13	31.7%	2	7.4%	
3	18	11.0%	6	14.6%	0	0.0%	
4	24	14.7%	1	2.4%	0	0.0%	
cGvHD (yes), *n* (%)	for 157 pt.						**<0.001**
	120	76.4%	21	51.2%	7	25.0%	
cGvHD grade, *n* (%)	for 157 pt.						**<0.001**
0	37	23.6%	20	48.8%	21	75.0%	
1	49	31.2%	7	17.1%	2	7.1%	
2	71	45.2%	14	34.1%	5	17.9%	
Karnofsky scale at the day of last contact, Me [Q1; Q3]	85 [0; 100]		90 [80; 100]		NA		1.000
Patient status at the day of last contact, *n* (%)							
Dead	76	44.4%	16	39.0%	8	29.6%	0.268
Relapse	73	42.7%	14	35.9%	3	11.1%	**0.007**

**Table 3:** Results of multivariate analysis based on patient, disease, and transplant characteristics for patients for all cohorts. Statistically significant results are highlighted in bold. Note: only statistically significant results were included. The full results of the multivariate analysis are included as Supplementary Materials. Abbreviations: GvHD—graft-versus-host disease, ATG—anti-thymocyte globulin, CML—chronic myeloid leukemia, RIC—reduced intensity conditioning.

Variable	Risk FACTOR for	HR	95% CI	*p* Value
Donor sex: female	Acute GvHD	**2.17**	**1.15–4.08**	**0.017**
GvHD prophylaxis: ATG		**2.80**	**1.15–6.79**	**0.023**
Donor sex: female	Chronic GvHD	**2.43**	**1.26–4.69**	**0.009**
RIC conditioning		**0.24**	**0.11–0.51**	**<0.001**
CML accelerated phase		**3.13**	**1.05–9.36**	**0.041**

**Table 4:** Survival outcomes. The comment “for (number) pt.” means the number of patients for whom data were available. Abbreviations: IMA—imatinib, 2G-TKI—2nd generation tyrosine kinase inhibitor, OS—overall survival. Statistically significant results are highlighted in bold.

	TKI-Naïve *n* = 171	IMA *n* = 41	2G-TKI *n* = 28	*p*-Value
Median follow-up time, months	41 [28; 73] for 163 pt.	19 [14; 24]	30 [16; 40]	**<0.001**
3-year overall survival OS (t = 3 years)	55%	49.9%	69.6%	0.740

## Data Availability

The authors confirm that the data supporting the findings of this study are available within this article.

## Supplementary Materials

The following supporting information can be downloaded at https://www.mdpi.com/article/10.3390/biomedicines13010163/s1, Table S1: Results of logistic regression analysis—aGvHD > 0; Table S2: Results of logistic regression analysis—cGvHD ≥ 1.
