# Supplementary material for: Impact of First- and Second-Generation Tyrosine Kinase Inhibitors on the Development of Graft-Versus-Host Disease in Individuals with Chronic Myeloid Leukemia: A Retrospective Analysis on Behalf of the Polish Adult Leukemia Group
Source: Biomedicines. 2025 Jan 11;13(1):163. doi: 10.3390/biomedicines13010163 (PMC11759780; doi:10.3390/biomedicines13010163)
Supplement: Supplementary file 1 [file biomedicines-13-00163-s001.zip › biomedicines-3382041-supplementary.pdf]

Table S1. *Results of logistic regression analysis - aGvHD >0.*

Risk factors for aGvHD>0	*b*	*p*	*beta*	*p*	*OR* (95% CI)
PA	-0.024	0.077	0.008	0.629	1.01 (0.98-1.04)
DA	-0.009	0.541	-	-	-
MTX	0.356	0.293	-	-	-
PM	0.251	0.356	-	-	-
DF	**0.651**	**0.028**	**0.773**	**0.017**	**2.17 (1.15-4.08)**
PCMV	0.147	0.701	-	-	-
DCMV	**-0.706**	**0.038**	-0.466	0.276	0.63 (0.27-1.46)
SIB	-0.546	0.058	-	-	-
MUD	**0.651**	**0.028**	-0.083	0.855	0.92 (0.38-2.25)
PBSC	-0.051	0.851	-	-	-
BMT	0.051	0.851	-	-	-
RIC	-0.576	0.105	-0.764	0.088	0.47 (0.19-1.12)
ATG	**1.083**	**0.001**	**1.030**	**0.023**	**2.80 (1.15-6.79)**
cCML	-0.644	0.066	-0.643	0.143	0.53 (0.22-1.24)
aCML	0.426	0.343	-	-	-
bCML	0.500	0.556	-	-	-
sCML	0.800	0.175	0.178	0.807	1.20 (0.28-5.04)
INFα	0.706	0.076	0.132	0.768	1.14 (0.47-2.76)

Table S2. *Results of logistic regression analysis - cGvHD ≥ 1.*

Risk factors for grade cGvHD ≥ 1	*b*	*p*	*beta*	*p*	*OR* (95% CI)
PA	-0.015	0.265	-	-	-
DA	-0.003	0.828	-	-	-
MTX	-0.031	0.935	-	-	-
PM	0.229	0.418	-	-	-
DF	**0.895**	**0.006**	**0.887**	**0.009**	**2.43 (1.26-4.69)**
PCMV	0.607	0.118	-	-	-
DCMV	0.184	0.574	-	-	-
SIB	0.539	0.064	-	-	-
MUD	-0.530	0.072	-	-	-
PBSC	-0.445	0.116	-	-	-

BMT	0.445	0.116	-	-	-
RIC	**-1.371**	**<0.001**	**-1.424**	**<0.001**	**0.24 (0.11-0.51)**
ATG	-0.280	0.332	-	-	-
cCML	0.044	0.895	-	-	-
aCML	0.881	0.091	**1.142**	**0.041**	**3.13 (1.05-9.37)**
bCML	-0.960	0.218	-	-	-
sCML	-0.700	0.158	-	-	-
INFα	0.111	0.771	-	-	-

Abbreviations
The continuous quantitative independent variables were:
PA – Recipient's age (from 15 to 68 years)
DA – Donor's age (from 15 to 68 years)

The discrete quantitative independent variable was:
MTX – Number of MTX doses (0, 1, 2, 3, 4)

The dichotomous qualitative independent variables were:
PM – Male gender of the recipient
DF – Female gender of the donor
PCMV – Positive CMV status of the recipient
DCMV – Positive CMV status of the donor
SIB – Type of transplant: sibling
MUD – Type of transplant: matched unrelated donor
PBSC – Cell source: peripheral blood stem cell
BM – Cell source: bone marrow
RIC – Type of conditioning: reduced-intensity conditioning (RIC)
MAC – Type of conditioning: myeloablative conditioning (MAC)
ATG – Use of ATG in GvHD prophylaxis
cCML – Chronic phase of CML at the time of transplant
aCML – Accelerated phase of CML at the time of transplant
bCML – Blast crisis phase
sCML – Second or subsequent chronic phase
INFα – Use of INF alpha
